# Estrogen Aggravates Tumor Growth in a Diffuse Gastric Cancer Xenograft Model

**DOI:** 10.3389/pore.2021.622733

**Published:** 2021-04-16

**Authors:** Sunyi Lee, Kyoung Mee Kim, Seung Yeon Lee, Joohee Jung

**Affiliations:** ^1^ Duksung Innovative Drug Center, Duksung Women’s University, Seoul, Korea; ^2^ College of Pharmacy, Duksung Women’s University, Seoul, Korea

**Keywords:** diffuse gastric cancer, SUN-16 cells, estrogen, ovariectomized mice, cancer xenograft model

## Abstract

Gastric cancer has the fifth-highest incidence rate and is the third leading cause of cancer-related deaths worldwide. The incidence of gastric cancer is higher in men than in women, but for the diffuse types of gastric cancer, the trend is opposite. Estrogen is considered the prime culprit behind these differences. Nevertheless, the action of estrogen in gastric cancers remains unclear. In this study, we investigated the effect of estrogen on diffuse-type gastric cancer. Human female diffuse gastric cancer SNU-16 cells were transplanted into male and female mice to analyze the effect of endogenous estrogen on tumor growth. Furthermore, the effect of exogenous estrogen was evaluated in ovariectomized mice. Expressed genes were compared between female and male xenograft models using RNA sequencing analysis. Furthermore, human gene expression omnibus databases were utilized to examine the effect of our target genes on overall survival. SNU-16-derived tumor growth was faster in female mice than in male mice. In total RNA sequencing, interferon gamma receptor 2 (*IFNGR2*), IQ motif containing E (*IQCE*), transient receptor potential cation channel subfamily M member 4 (*TRPM4*), and structure-specific endonuclease subunit SLX4 (*SLX4*) were found. These genes could be associated with the tumor growth in female diffuse-type gastric cancer which was affected by endogenous estrogen. In an ovariectomized gastric cancer xenograft model, exogenous estrogen promoted tumor growth. Especially, our results indicated that estrogen induced G protein-coupled estrogen receptor expression in these mice. These results suggest that estrogen aggravates tumor progression in female diffuse gastric cancer.

## Introduction

In the year 2018, gastric cancer had the fifth-highest incidence rate and was the third leading cause of cancer-related deaths worldwide [1]. Many researchers are closely investigating the characteristics of gastric cancer cells to identify potential biomarkers for enhanced diagnostic and therapeutic approaches. Interestingly, gastric cancer incidence and mortality rates seem to differ markedly based on sex [1]. Nevertheless, studies that consider sex-based differences in gastric cancer are rare; *in vivo* systems such as xenograft mouse models provide a convenient approach to study these sex-based differences.

The incidence or prognosis varies depending on the type of gastric cancer. Based on molecular characterization, gastric adenocarcinomas were divided into four subtypes as follows: tumors positive for Epstein-Barr virus (EBV), microsatellite unstable tumors (MSI), genomically stable tumors (GS), and tumors with chromosomal instability (CIN) [2]. The incidence rate is higher in males than in females, except for with MSI type [3]. Gastric cancers are histologically classified as intestinal types (papillary, well-differentiated, moderately differentiated, and mucinous types) and diffuse types (poorly-differentiated, signet ring cell, and undifferentiated types) according to Lauren [4]. Chandanos et al. reported that estrogen protects gastric adenocarcinoma of the intestinal type; however, the diffuse type occurs more in premenopausal women than in postmenopausal women or men [5]. In Korea, young women patients with gastric cancer almost all have the diffuse type [6]. The most evident cause of sex-based differences is sex hormones. In this study, we focused on the effect of estrogen on gastric cancer progression. Different studies have reported that estrogen affects the progression of breast, liver, and lung cancers [7–11]. Evidently, menopause was noted to be a turning point for this phenomenon, as a low estrogen/androgen ratio becomes apparent. Based on several studies, the risk of liver [12] and gastric [13] cancers in postmenopausal women is similar to that in man. Thus, the effect of estrogen on cancer progression is important to establish an anticancer strategy. To date, the effect of hormone replacement therapy on gastric cancer patients has been controversial [14, 15].

Mechanistically, estrogen binds to estrogen receptors (ERs) and acts through various downstream signaling pathways; however, the different ER subtypes (ERα and ERβ) were reported to yield paradoxical results. Takano et al. correlated *ER* mRNA expression in gastric cancer tissues with poor prognosis and metastasis [16]. The expression of ER is associated with a poor diagnosis for chemotherapy after operation in gastric cancer [17]. However, Qin et al. reported that ERα expression enhances apoptosis in gastric cancer MKN28 cells [18]. Futher, Chandanos et al. reported that ERβ levels are lower in gastric adenocarcinoma tissue than in the non-tumor gastric mucosa region [5]. Furthermore, the G protein-coupled estrogen receptor (GPER) also plays a role in mediating the effects of estrogen, thereby inducing the activation of non-genomic signaling pathways. GPER was reported to induce invasion and proliferation in breast and ovarian cancers; however, it was found to have a cancer-suppressive effect in non-small cell lung cancer, liver cancer, and triple negative breast cancer [19–21]. Nonetheless, studies regarding the role of ERs in gastric cancer progression are limited, which motivated us to investigate this matter.

In this study, the effect of estrogen on the diffuse type of gastric cancer was comprehensively studied using SNU-16 cells. To elucidate the role of estrogen, we utilized a variety of approaches including a xenograft mouse model and a bioinformatics analysis and considered hormonal changes in menopause.

## Materials and Methods

### Cell Culture

Human gastric cancer SNU-16 cells (No. 00016), SNU-620 cells (No. 00620), and SNU-484 cells (No. 00484) were purchased from the Korean Cell Line Bank (KCLB, Seoul, Korea). These cells were maintained in RPMI-1640 medium (GenDEPOT, Barker, TX) supplemented with 10% fetal bovine serum (Young In Frontier, Seoul, Korea) and 1% penicillin-streptomycin (GenDEPOT). The cells were cultured in a 5% CO_2_ humidified incubator at 37°C.

### Cell Viability

The cells (1 × 10^4^ cells/well) were seeded into 96-well plates. Ten nanomolar of 17β-estradiol (E2758, Sigma-Aldrich, Inc., MO, United States) in 0.1% ethanol was administered to the cells for 24 or 48 h. A solvent as 0.1% ethanol was applied to control cells. The cell numbers were determined by methyl thiazolyl tetrazolium (MTT) cell proliferation assays [22]. Briefly, 20 μl of MTT (5 mg/ml, Sigma-Aldrich, Inc.) solution was added to each well and incubated for 3 h at 37°C. Thereafter, the media were removed and 150 μl of dimethyl sulfoxide (Sigma-Aldrich, Inc.) was added into each well for 30 min. Absorbance was measured using a microplate reader (Infinite M200 PRO, TECAN, Switzerland) at 560 nm. MTT assay was repeated twice. Data were represented as the mean ± standard deviation (*n* = 6).

### Animals and Experimental Conditions

All animal experiments were approved by the Institutional Animal Care and Use Committee of Duksung Women’s University (No. 2016-003-004) in accordance with the guidelines for the care and use of laboratory animals. Male and female NCr nude mice (CrTac:NCr-Foxn1nu, 5-weeks-old) were purchased from Nara Biotech (Gyeonggi, Korea). The animals were left to acclimatize for 1 week prior to any procedural work and kept at optimal conditions (20°C, 50% humidity, and a 12/12-h light/dark cycle). Diet was provided with drinking water ad libitum.

### Establishment of the Xenograft Model and Measurement of Tumor Growth

To generate a xenograft model, SNU-16 cells (5 × 10^6^ cells/mice) were subcutaneously injected into the right hind legs of female and male NCr nude mice (*n* = 5/group) [23]. This experiment was repeated twice. Tumor sizes were measured three times per week using calipers. Moreover, the animals were weighed and monitored regularly for signs of distress. Tumor volume was calculated using the following equation:

$$\mathrm{Tumor}\;\mathrm{volume}\left(mm^{3}\right)=\left(\mathrm{the}\;\mathrm{longest}\;\mathrm{length}\right)\times {\left(\mathrm{the}\;\mathrm{shortest}\;\mathrm{length}\right)}^{2}/2$$ (1)

Data are presented as the mean ± standard deviation.

### Ovariectomy

Female NCr nude mice (8-weeks-old) were purchased from Nara Biotech. The animals were first anesthetized before a 1-cm incision was made on their dorso-lateral abdominal walls through which the ovaries were removed [24]. After confirming that there was no bleeding, the surgical wounds were closed. To allow the uterus to regress to a minimum, stable baseline, a time of 14 days elapsed. Then, SNU-16 cells (5 × 10^6^ cells/mice) were transplanted into the right hind legs of these mice and tumor growth was measured three times per week.

### Implantation of Estrogen Pellet

After the tumor was visually checked in the ovariectomized xenograft model, the groups were randomly divided. Then, an estrogen pellet (Innovative research of America, FL, United States) was implanted into a subcutaneous pocket on the dorsal flank of mice. Estrogen was consistently released at 8.3 μg/day from the pellet for 60 days [23]. To confirm estrogen effect, mice was anesthetized by isoflurane (Forane, JW Pharmaceutical, Seoul, Korea) after the final tumor volume measurement. The blood was collected from abdominal vein of anesthetized mice and the uterus was isolated. The blood was leaved at room temperature for 30 min, and then the clot was removed by centrifuging at 3,000 rpm for 10 min in 4°C. The supernatant as a serum was taken to a new tube. Serum estrogen level was measured with electrochemiluminescence immunoassays on Roche Cobas 8,000 analyzer (Roche Diagnostics, Basel, Switzerland) by DKKorea (Seoul, Korea) [25]. The uterus weight was measured using an electronic weighing balance (OHAUS Instruments, Shanghai, China).

### Total RNA Sequencing and Analysis

After the final tumor measurement, the SNU-16-derived tumor tissues were isolated from sacrificed mice, immersed, and stored in liquid nitrogen until analysis. Total RNA from the tumor tissues was extracted with the RNeasy Mini Kit (Qiagen, MD, United States) according to the manufacturer’s instructions. In brief, 30 mg of frozen tumor tissues were submerged in 600 μl of buffer RLT and then immediately homogenized using Tissueruptor II (Qiagen) on ice for 30 s. The lysate was centrifuged at 15,000 rpm for 3 min in 4°C. Ethanol was added to the supernatant (lysate). The lysate was loaded into an RNeasy mini spin column. Contaminants including DNA were removed, and then RNA was eluted in water. Then, the quality and concentration of the RNA samples were determined based on an electropherogram and RNA integrity was calculated using the Agilent 2,100 BioAnalyzer (Agilent, CA, United States). Samples with an RNA integrity number greater than six were sequenced using an Illumina HiSeq 2,500 (Illumina, CA, United States). Based on the fragments per kilobase million (FPKM) value of each gene, differentially expressed genes (DEGs) were selected. DEG analysis was performed using the Cuffdiff tool with *p*-value ≤ 0.00005 and q-value (a multiple-test corrected *p*-value) ≤ 0.05, whereas the cut-off for the gene ontology analysis was *p* < 0.0001 [26, 27].

### Analysis of Genomic Data from Gene Expression Omnibus Database

Publicly available data (Supplementary Table S1) in Kaplan-Meier Plotter (kmplot.com) were used in this study; patient clinical data were obtained from the Gene Expression Omnibus database (GEO database: GSE15459, GSE22377, GSE29272, GSE38749, and GSE62254 datasets). The data contained mRNA expression profiles of 112 female patients and 127 male patients with diffuse type-gastric cancer based on microarray. Overall survival (OS) analysis was shown as a Kaplan-Meier curve [28]. Data were divided into two groups based on the gene expression levels. A cut-off refers to the best performing threshold. The hazard ratios (HR) with 95% confidence intervals and *p*-values were automatically calculated in Kaplan-Meier Plotter.

### Western Blotting Analysis

Tumor tissues were lyzed in radioimmunoprecipitation assay buffer (RIPA buffer, GenDEPOT) with Xpert protease inhibitor cocktail solution (P3100, GenDEPOT) and Xpert phosphatase inhibitor cocktail solution (P3200, GenDEPOT). The proteins (10 μg) were separated by 10% or 12% SDS-polyacrylamide gel electrophoresis and transferred to polyvinylidene fluoride membranes (Millipore, Darmstadt, Germany). The membranes were then blocked with 5% skimmed milk in TBST (Tris-buffered saline with 0.1% tween 20) and incubated with primary antibodies for artemin (GeneTex, CA, United States, 1:1,000), ERα (Santa Cruz Biotechnology, sc-130072, 1:1,000), ERβ (Santa Cruz Biotechnology, sc-53494, 1:1,000), GPER (Abcam, ab188999, Cambridge, United Kingdom, 1:1,000) and β-actin (Sigma-Aldrich, Darmstadt, Germany, 1:5,000) overnight at 4°C. The next day, the membranes were incubated with secondary antibodies (goat anti-mouse IgG-HRP conjugated or goat anti-rabbit IgG-HRP conjugated, 1:3,000) for 3 h at room temperature. Proteins were visualized using enhanced chemiluminescent solution and detected with a Chemi-Doc (FluorChem E system, San Jose, California, United States).

### Statistical Analysis

Statistical analysis was performed using GraphPad Prism 7 (GraphPad Software Inc., CA, United States). The data were analyzed using Student’s t-test or one-way ANOVA test followed by Tukey’s multiple comparisons test as post hoc test. Significant differences were assumed for *p*-values less than 0.05.

## Results

### SNU-16 Cell-Derived Tumors Progress Faster in Female Mice

**FIGURE 1:**
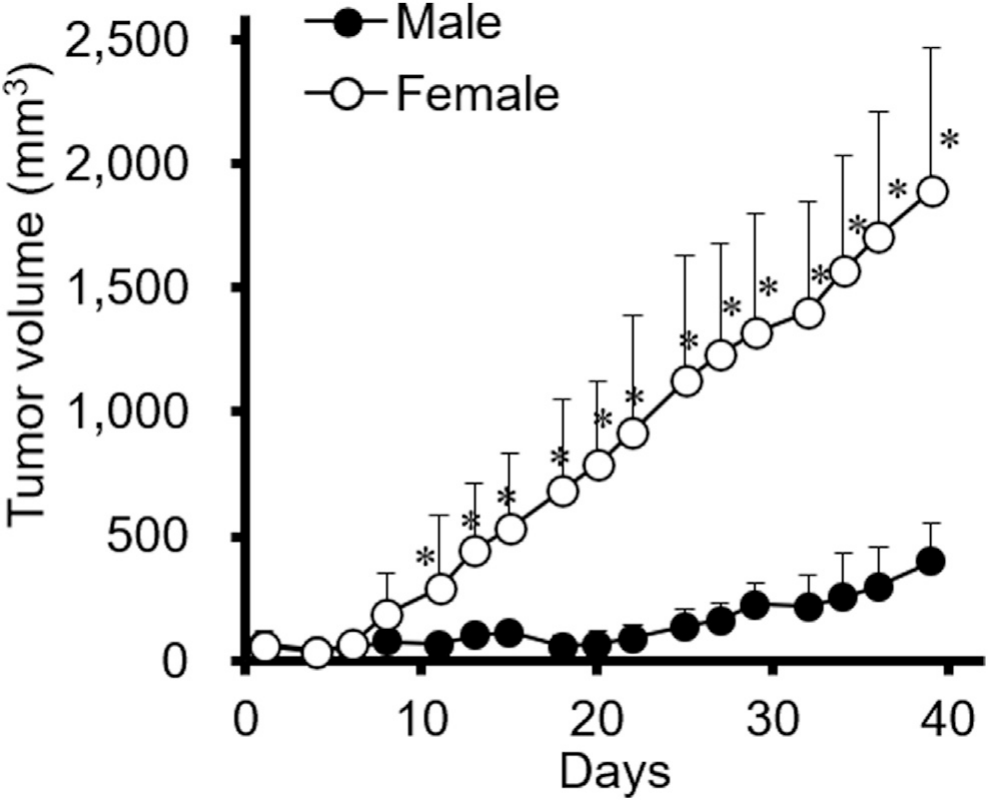
The effect of sex on SNU-16-tumor growth in a xenograft mouse model. SNU-16 cells were injected into female and male NCr nude mice. SNU-16-derived tumor volume was calculated as indicated in the Materials and Methods. All data are presented as the mean ± standard deviation (SD; *n* = 5/group); *, *p* < 0.05 (Student’s *t*-test).

Xenograft mouse models were established to investigate whether tumor progression varied depending on sex differences. The female gastric cancer cell line SNU-16 was used to induce tumors in female and male mice and the resulting tumors were examined; as shown in Figure 1, tumor progression was significantly faster in female mice than in male mice. It was further investigated whether other diffuse-type gastric cancer cells are affected by the sex of mice. As shown in Supplementary Figure S1A, results of SNU-620 female diffuse gastric cancer cells were in accordance with those of SNU-16 cells although the statistical significance was not shown. However, SNU-484 male diffuse gastric cancer cells showed no difference in tumor growth between male and female xenograft models (Supplementary Figure S1B). Only female-originating cancer cells showed enhanced tumor growth in female mice. To elucidate the involvement of female sex hormones in tumor progression, the expression level of ERs was investigated in these gastric cancer cell lines. There was no difference in the expression level of ERs according to sex or cancer type in gastric cancer cell lines (Supplementary Figure S2). These results suggested that endogenous factors in female mouse xenograft models affect tumor growth rather than a direct ER-mediated pathway in female diffuse-type gastric cancer cells.

### Differentially Expressed Genes from Total RNA Sequencing Affect the Overall Survival of Women Patients with Diffuse-Type Gastric Cancer

**FIGURE 2:**
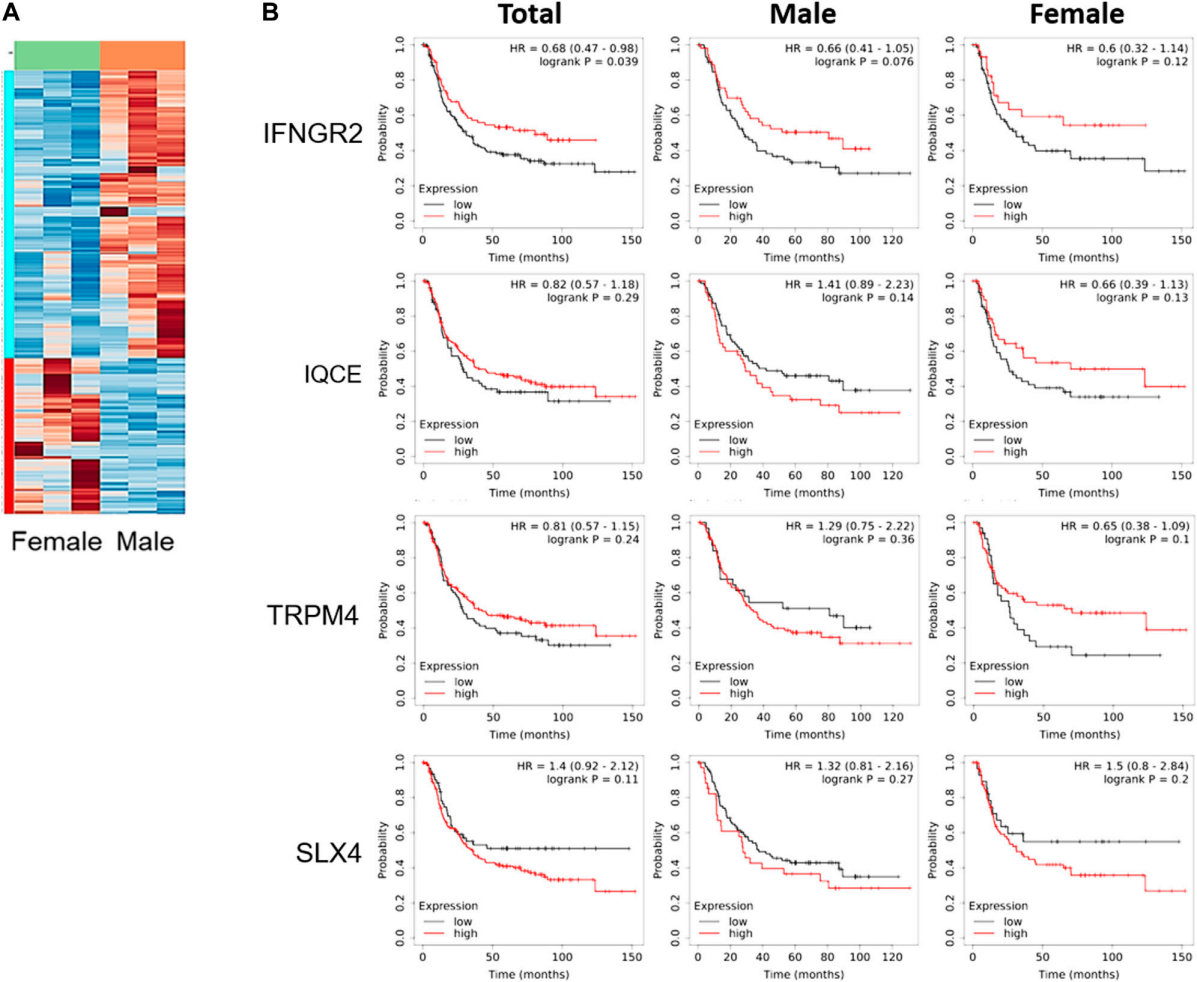
Clinical survival curve of diffuse-type gastric cancer patients depending on differentially expressed genes. **(A)** Heatmap of gene expression based on total RNA seq of SNU-16-derived tumor tissues (*n* = 3/group), **(B)** Kaplan-Meier curve of differentially expressed genes obtained from gene expression omnibus database (Total: *n* = 239; men: *n* = 127; women: *n* = 112). HR, the hazard ratio with 95% confidence intervals.

To better understand the difference reported in Figure 1, tumor tissues obtained from these xenograft models were compared using total RNA sequencing (RNA seq). The data of RNA seq were analyzed based on human-originating genes excluding mouse-originated genes. We observed DEGs between female and male mice (Figure 2A); transcripts of DEGs with a log_2_fold_change (fc) > absolute value of 1.5, *p*-values ≤ 0.00005, and q-values ≤ 0.05 are summarized in Table 1. As shown in Table 1, interferon gamma receptor 2 (*IFNGR2*) had lower expression levels in tissues from female mice than in male tissues, whereas IQ motif containing E (*IQCE*), transient receptor potential cation channel subfamily M member 4 (*TRPM4*) and structure-specific endonuclease subunit SLX4 (*SLX4*) exhibited higher expression in females than in males. To investigate the association between our *in vivo* findings and clinical data, we compared genes obtained from our RNAseq data with those from clinical databases. As shown in Figure 2B, the OS of patients with diffuse-type gastric cancer obtained from the GEO database was reanalyzed depending on expression levels of DEGs. There is a trend toward better survival at high *IFNGR2*, *IQCE*, and *TRPM4* expression levels and at low *SLX4* expression level but cannot claim there was any association in women. Only *IFNGR2* has a positive prognostic value, and only among all patients.

**TABLE 1:** Difference of expressed genes between male and female xenograft model.

Gene name	FPKM^a^		Log_2_fc^b^	*p*-value
	Male	Female		
*IFNGR2*	24.7	5.06	−2.29	0.00005
*IQCE*	1.05	3.83	1.87	0.00005
*TRPM4*	3.48	12.4	1.83	0.00005
*SLX4*	0.299	0.852	1.51	0.00005

^a^ Fragments per kilobase of exon per million reads (mean value, *n* = 3).

^b^ fc: fold change = the mean expression value in female group/the mean expression value in male group.

### Estrogen Might Indirectly Affect the Proliferation of SNU-16 Cells

**FIGURE 3:**
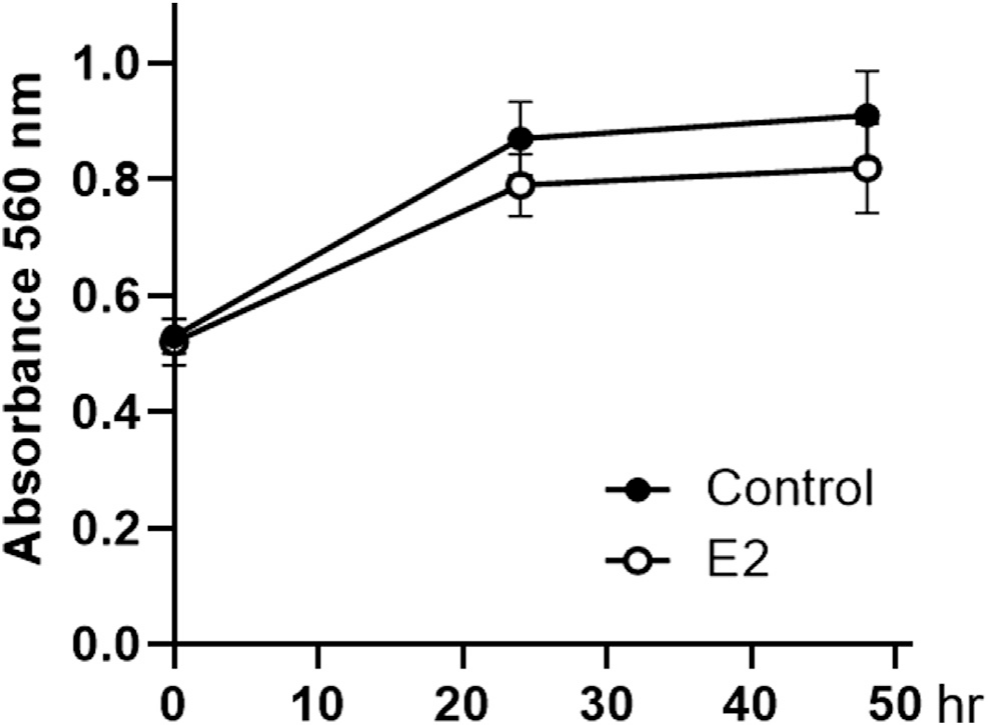
Effect of 17β-estradiol on the proliferation of SNU-16 cells. Cell proliferation of 17β-estradiol (E2, 10 nM)-treated SNU-16 cells; data are presented as the mean ± SD (*n* = 6).

Considering that SNU-16-derived tumors progressed faster in female mice than in male mice during the evaluation of cancer xenograft models (Figure 1), we investigated whether estrogen could directly affect the proliferation of these cells. As shown in Figure 3, cell proliferation was stagnant after 24 h of 10 nM of 17β-estradiol (E2 as estrogen) treatment, but there was no significant difference compared to that in the control group.

### Exogenous Estrogen Promotes Diffuse-type Gastric Cancer Progression in Cancer Xenograft Models of Ovariectomized Mice

**FIGURE 4:**
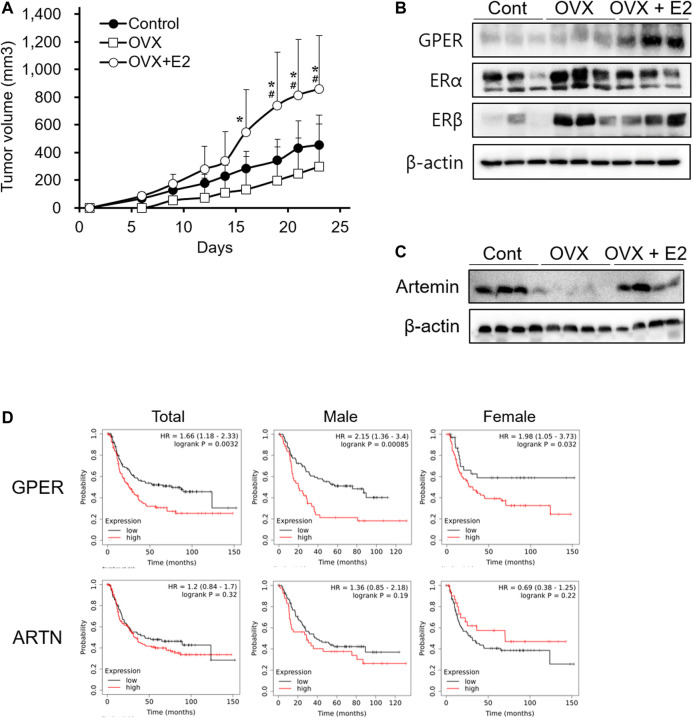
Enhancement of tumor growth mediated by 17β-estradiol-induced GPER and ARTN in an ovariectomized xenograft mouse model. **(A)** Effect of 17β-estradiol (E2) on SNU-16-derived tumor growth in ovariectomized mice (OVX); data are presented as the mean ± SD (*n* = 5/group). Significant differences were considered depending on *p* values, as calculated by one-way ANOVA followed by Tukey’s post hoc test; * (OVX vs OVX + E2), # (Control vs OVX + E2), *p* < 0.05, **(B)** Protein expression levels of estrogen receptors in SNU-16-derived tumor tissues by western blotting, **(C)** Protein expression levels of artemin (ARTN) in SNU-16-derived tumor tissues by western blotting, **(D)** Kaplan-Meier curve based on GPER and ARTN obtained from the gene expression omnibus database (number of men with diffuse-type gastric cancer = 127; number of women with diffuse-type gastric cancer = 112). HR, the hazard ratio with 95% confidence intervals.

*In vitro*, E2 showed no direct effect on cell proliferation (Figure 3). Therefore, we investigated whether E2 deficiency and exogenous E2 treatment could affect tumor progression in xenograft models. Ovariectomized mice were used to better understand the factors involved during tumor progression in the female xenograft model. To ensure an accurate comparison, we confirmed that estrogen levels in the blood and the uterus weight were decreased in ovariectomized mice, and then estrogen treatment was restored (Supplementary Figure S3). In the ovariectomized xenograft mouse (OVX) model, SNU-16-derived tumor growth was detected (Figure 4A). The OVX group showed slightly delayed tumor growth compared to the normal xenograft group (control group). However, no significant difference between the two groups was noted. Interestingly, the estrogen-treated group (OVX with E2 group) had significantly enhanced tumor growth when compared to that in either OVX or control groups (Figure 4A). To elucidate the mechanism of estrogen-induced tumor growth, the expression of ER subtypes was determined by western blotting (Figure 4B). ERα and ERβ expression levels were slightly increased in ovariectomized mice to generate a sensitive response to low estrogen levels. Surprisingly, GPER expression levels were induced in the OVX with E2 group more than in the OVX group. These results suggested that E2 might affect tumor growth through the regulation of ER expression levels, and especially GPER. To elucidate the effect of E2 on SNU-16-derived tumor growth in ovariectomized mice, we performed RNA seq and summarized DEGs among these groups (Supplementary Table S2). Among genes expressed only in OVX with E2 group, artemin (ARTN) was known as a factor involved in the cancer progression [29]. Thus, we first investigated the change in the level of ARTN expression by western blotting. ARTN levels were significantly decreased in the OVX group; however, E2 treatment significantly induced ARTN expression (Figure 4C). Based on the Kaplan-Meier curve using the GEO database, the OS of diffuse-type gastric cancer patients showed a negative association with *GPER* expression levels. A trend of OS with *ARTN* expression was shown, but no statistically effects were measured (Figure 4D).

## Discussion

In this study, we investigated the effect of endogenous and exogenous estrogen on diffuse-type gastric cancer xenograft models. Generally, the incidence rate of gastric cancer is higher in males than in females. However, diffuse-type gastric cancer that expresses ER has a high incidence rate and is associated with poor prognosis in young women [5]. SNU-16 cells are known as a diffuse-type gastric cancer cell line derived from a 33 year-old woman [30]. Our results demonstrated that SNU-16-derived tumors also showed higher growth in female mice than in male mice, similar to clinical data (Figure 1). These results agreed with the findings of a previous study, which showed that sex-specific xenograft models are crucial to more accurately reflect tumor progression and drug sensitivity [31]. Moreover, we established an ovariectomized xenograft model to investigate the effect of E2 on the growth of cancer cells. E2 accelerated the growth of gastric cancer after ovariectomy (Figure 4A). The OVX model could be assumed to represent postmenopausal gastric cancer patients. These results suggested that the control of E2 might be important for the therapeutic strategy for women patients with diffuse-type gastric cancer.

Environmental factors including E2 can regulate several genes that are related to the growth of SNU-16-derived tumors; here, we identified *IFNGR2, IQCE, TRPM4* and *SLX4.* IFNG plays crucial roles in the immune system, and thus, it is associated with viral infection and cancers. IFNG signaling is activated upon the binding of IFNGR, a heterodimer consisting of IFNGR1 and IFNGR2 [32]. Recently, Zaidi reported that IFNG has dual roles as a tumor suppressor and protumor factor in cancer [33]. IFNGR2 is a mediator of biological activities of IFNG. Thus, *IFNGR2* is a gene related to Th1 cell-mediated immune responses in gastric cancer. An *IFNGR2* polymorphism (Ex7-128 C > T) was reported to increase the risk of gastric cancer in a population-based study of Poland [34]. IQCE was reported to be a prognostic biomarker in endometrial cancer, and its level is positively associated with the OS rate of endometrial cancer patients [35]. TRPM4 is associated with proliferation, migration, and invasion of several cancer cells [36–39]. It is highly expressed in colorectal cancer and breast cancer [37, 38]. In endometrial carcinoma, decrease of its level shows aggressive cancer progression and a poor prognosis [40]. SLX4 is a DNA repair protein, and it plays a role of resistance to DNA damaging agents. Thus, SLX4 mutation (c.1114C > T) segregated along with familiar breast cancer gene and caused breast cancer susceptibility [41]. SLX4 is known as the most frequently mutated gene in Asian gastric cancer patients [42]. As mentioned above, these genes found in RNAseq were known to be associated with the OS or prognosis of various cancer types. However, in diffuse type gastric cancer, there was no significant difference in the association between the expression levels of these genes and OS.

The effect of exogenous E2 on diffuse-type gastric cancer was elucidated in an OVX xenograft model. Our results suggest that exogenous estrogen might promote tumor growth by inducing GPER and ARTN expression in OVX models (Figure 4). We found the GPER was a key factor in our results. GPER, a member of the G protein-coupled receptor (GPCR) family, was induced by E2 treatment in the OVX xenograft model. Similar to our results, the induction of GPER is also known to promote the proliferation of triple negative breast cancer [43]. Furthermore, we first identified *ARTN* from RNA seq data and observed that ARTN expression level increased when E2 treatment promoted tumor growth in ovariectomized mice. ARTN is a ligand that binds to various TGF-β receptors from the transforming growth factor (TGF)-β superfamily of proteins [44]. This ligand was also shown to promote tumor growth, metastasis, and drug resistance in mammary carcinoma [45]. Additionally, the mechanism underlying the effect of ARTN was found to be associated with the RET pathway and GPCR signaling pathways [46]. In the Kaplan-Meier curve using the GEO database, ARTN expression levels were not associated with OS of diffuse type gastric cancer, but high expression of GPER level was associated with worse OS in diffuse-type gastric cancer.

In conclusion, we suggest that sex differences should be considered in the evaluation of xenograft models. We presumed that estrogen might act indirectly through non-tumorous cells in the tumor mass. Especially, estrogen aggravates tumor proliferation in female diffuse-type gastric cancer xenograft models. Thus, female mice show faster tumor growth in a xenograft model bearing SNU-16 cells than male mice. Furthermore, E2 exposure stimulates SNU-16 derived tumor growth in ovariectomized mice. We also found that *IFNGR2*, *IQCE*, *TRPM4*, and *SLX4* could be associated with SNU-16 derived tumor growth in female mice by endogenous E2. Additionally, our results indicated that E2 induced GPER and ARTN expression and enhanced tumor growth in the ovariectomized gastric cancer xenograft model. These results suggest that the control of E2 could be an important target for female diffuse-type gastric cancer.

## Data Availability

The datasets presented in this study can be found in online repositories. The names of the repository/repositories and accession number(s) can be found in the article/Supplementary Material.
