# Supplementary material for: Estrogen Aggravates Tumor Growth in a Diffuse Gastric Cancer Xenograft Model
Source: Pathol Oncol Res. 2021 Apr 16;27:622733. doi: 10.3389/pore.2021.622733 (PMC8262185; doi:10.3389/pore.2021.622733)
Supplement: Supplementary file 1 [file Presentation1.PPTX]

## Slide 1
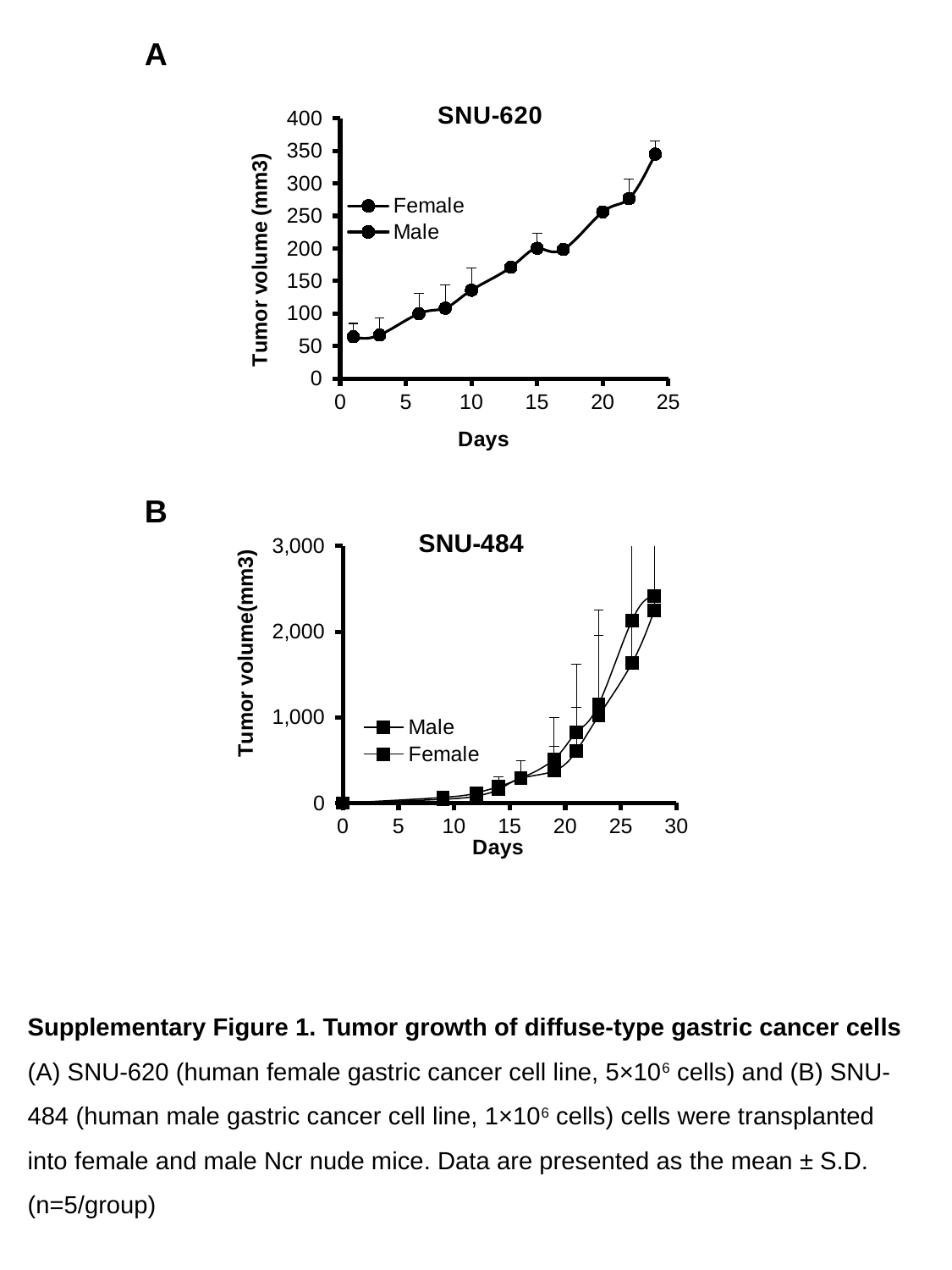

A
B
### Chart: SNU-620
| Category | Female | Male |
|---|---|---|
### Chart: SNU-484
| Category | | |
|---|---|---|Supplementary Figure 1. Tumor growth of diffuse-type gastric cancer cells
(A) SNU-620 (human female gastric cancer cell line, 5×10^6^ cells) and (B) SNU-484 (human male gastric cancer cell line, 1×10^6^ cells) cells were transplanted into female and male Ncr nude mice. Data are presented as the mean ± S.D. (n=5/group)

## Slide 2
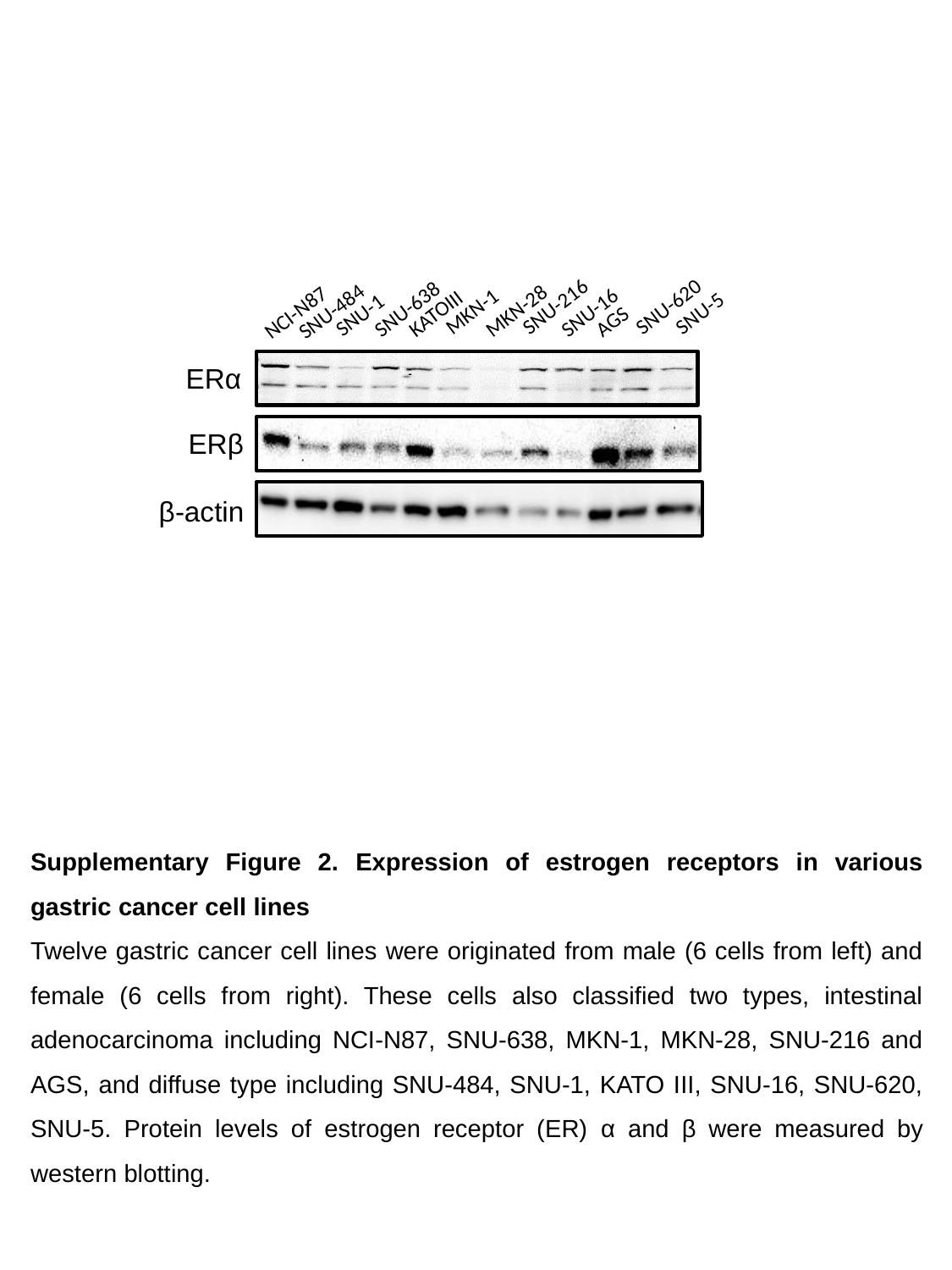

MKN-1
SNU-216
SNU-620
SNU-5
SNU-1
SNU-638
KATOIII
MKN-28
SNU-16
AGS
NCI-N87
SNU-484
ERα
ERβ
β-actin
Supplementary Figure 2. Expression of estrogen receptors in various gastric cancer cell lines
Twelve gastric cancer cell lines were originated from male (6 cells from left) and female (6 cells from right). These cells also classified two types, intestinal adenocarcinoma including NCI-N87, SNU-638, MKN-1, MKN-28, SNU-216 and AGS, and diffuse type including SNU-484, SNU-1, KATO III, SNU-16, SNU-620, SNU-5. Protein levels of estrogen receptor (ER) α and β were measured by western blotting.

## Slide 3
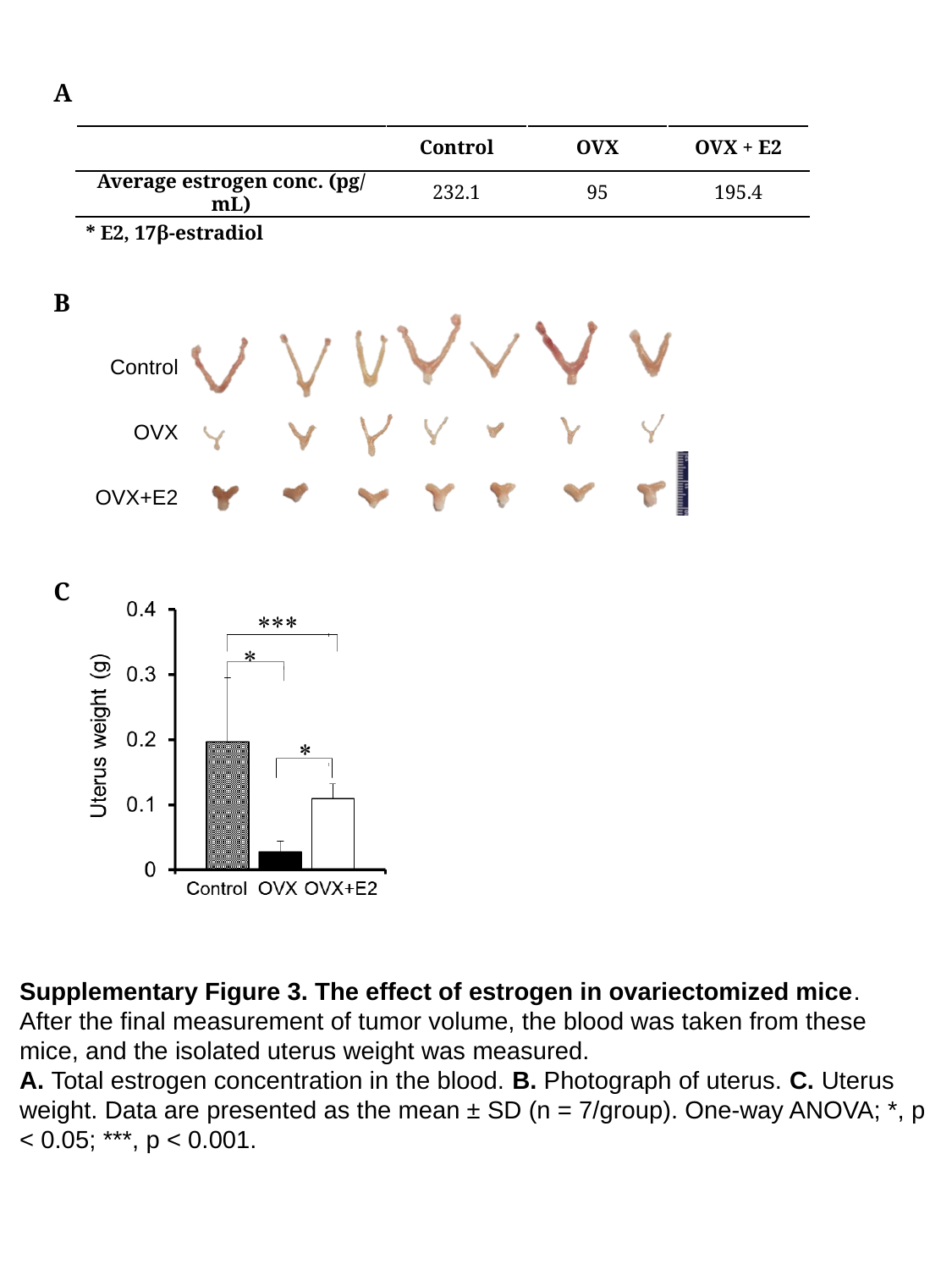

A
| | Control | OVX | OVX + E2 |
| --- | --- | --- | --- |
| Average estrogen conc. (pg/mL) | 232.1 | 95 | 195.4 |
| \* E2, 17β-estradiol | | | |
B
Control
OVX
OVX+E2
C
Supplementary Figure 3. The effect of estrogen in ovariectomized mice.
After the final measurement of tumor volume, the blood was taken from these mice, and the isolated uterus weight was measured.
A. Total estrogen concentration in the blood. B. Photograph of uterus. C. Uterus weight. Data are presented as the mean ± SD (n = 7/group). One-way ANOVA; *, p < 0.05; ***, p < 0.001.

## Slide 4
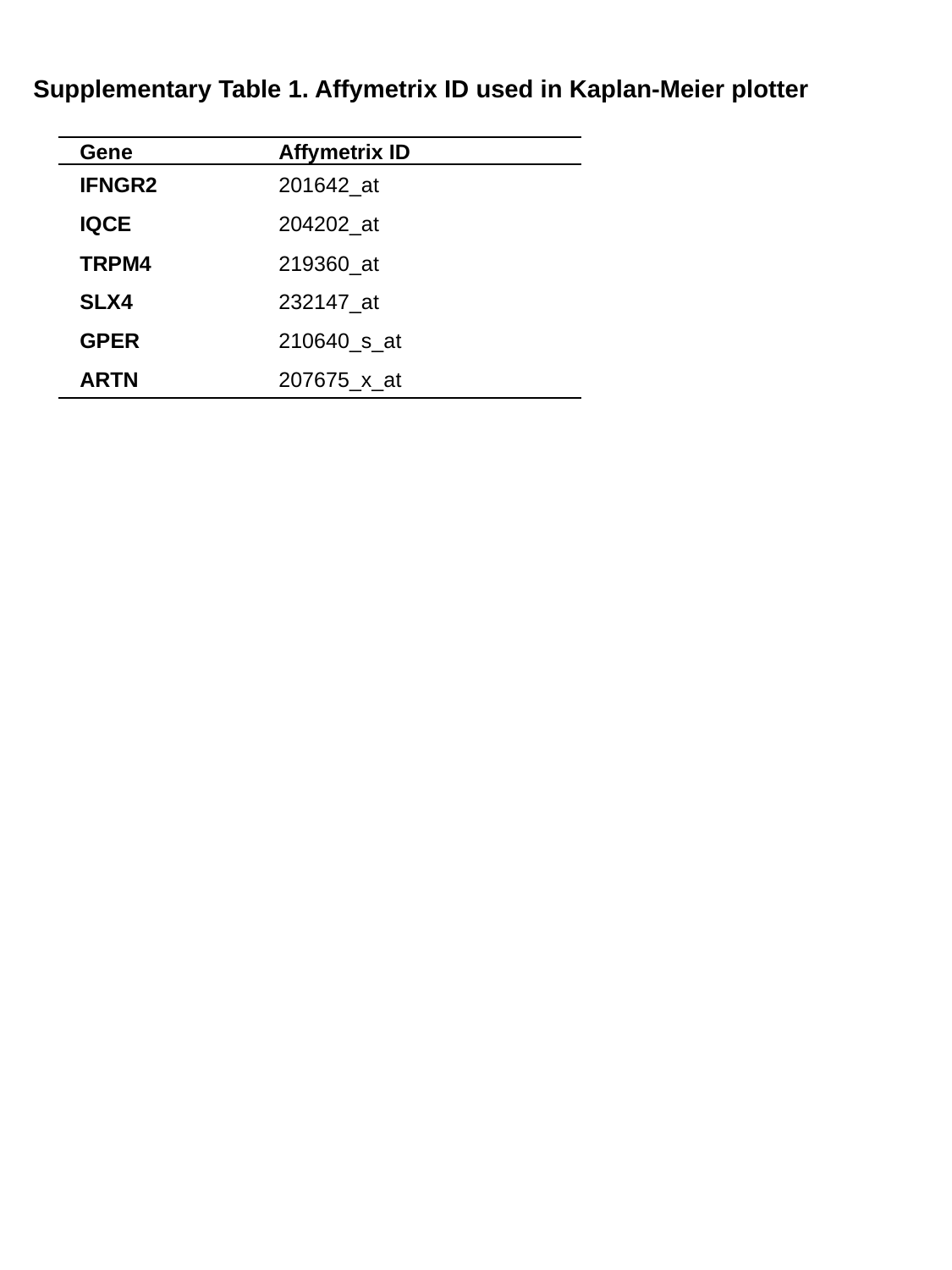

Supplementary Table 1. Affymetrix ID used in Kaplan-Meier plotter
| Gene | Affymetrix ID |
| --- | --- |
| IFNGR2 | 201642\_at |
| IQCE | 204202\_at |
| TRPM4 | 219360\_at |
| SLX4 | 232147\_at |
| GPER | 210640\_s\_at |
| ARTN | 207675\_x\_at |

## Slide 5
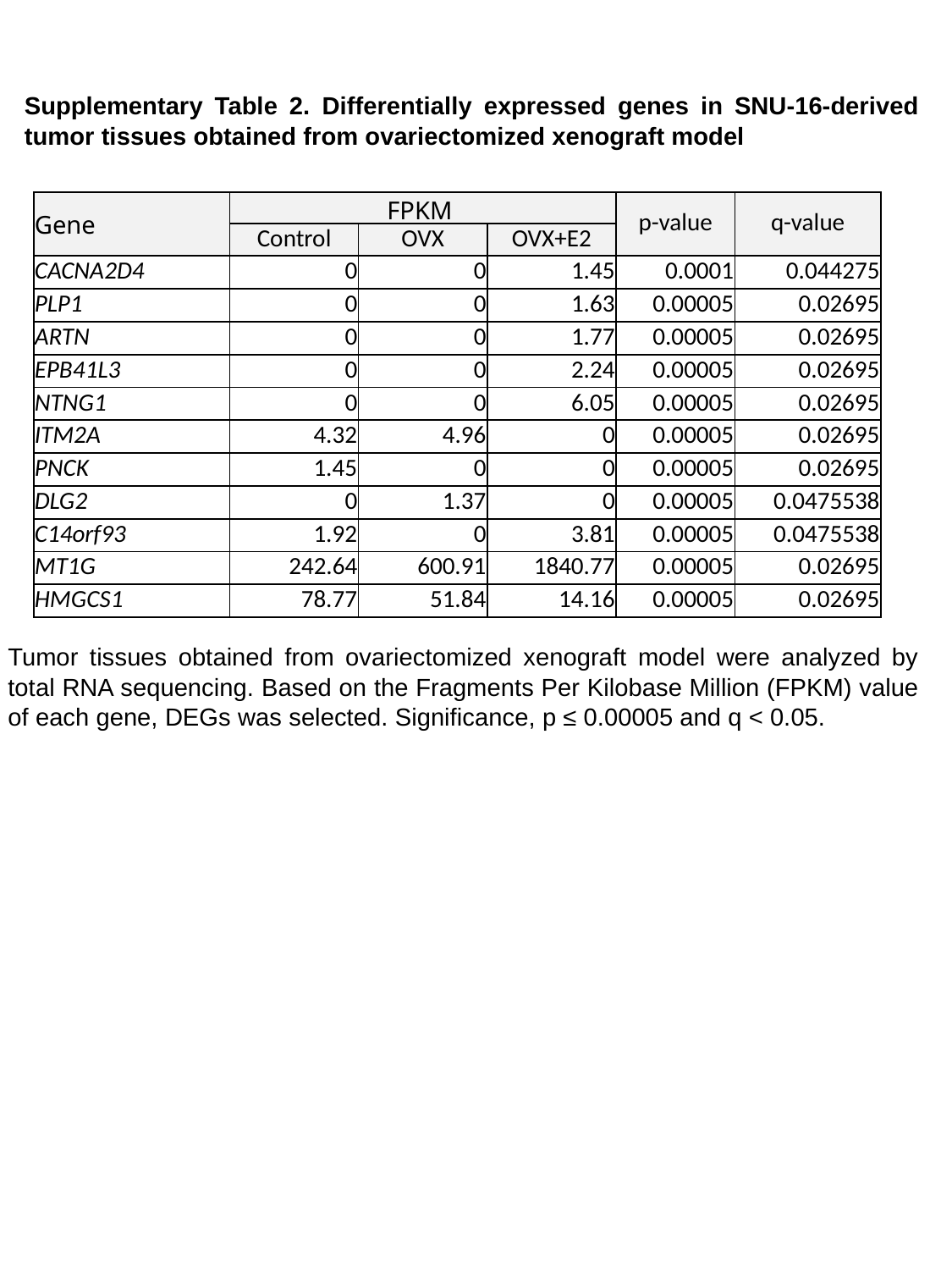

Supplementary Table 2. Differentially expressed genes in SNU-16-derived tumor tissues obtained from ovariectomized xenograft model
| Gene | FPKM | | | p-value | q-value |
| --- | --- | --- | --- | --- | --- |
| | Control | OVX | OVX+E2 | | |
| CACNA2D4 | 0 | 0 | 1.45 | 0.0001 | 0.044275 |
| PLP1 | 0 | 0 | 1.63 | 0.00005 | 0.02695 |
| ARTN | 0 | 0 | 1.77 | 0.00005 | 0.02695 |
| EPB41L3 | 0 | 0 | 2.24 | 0.00005 | 0.02695 |
| NTNG1 | 0 | 0 | 6.05 | 0.00005 | 0.02695 |
| ITM2A | 4.32 | 4.96 | 0 | 0.00005 | 0.02695 |
| PNCK | 1.45 | 0 | 0 | 0.00005 | 0.02695 |
| DLG2 | 0 | 1.37 | 0 | 0.00005 | 0.0475538 |
| C14orf93 | 1.92 | 0 | 3.81 | 0.00005 | 0.0475538 |
| MT1G | 242.64 | 600.91 | 1840.77 | 0.00005 | 0.02695 |
| HMGCS1 | 78.77 | 51.84 | 14.16 | 0.00005 | 0.02695 |
Tumor tissues obtained from ovariectomized xenograft model were analyzed by total RNA sequencing. Based on the Fragments Per Kilobase Million (FPKM) value of each gene, DEGs was selected. Significance, p ≤ 0.00005 and q < 0.05.
